# Open Osteochondral Fracture of the Lateral Trochlea in an Adult: The Role of Fibrocartilage

**DOI:** 10.7759/cureus.82360

**Published:** 2025-04-16

**Authors:** Georges F Bassil, Mohamed Jiddi, Zied Missaoui

**Affiliations:** 1 Orthopedics and Traumatology, Grand Hôpital de l'Est Francilien, Meaux, FRA

**Keywords:** fibrocartilage, herbert screw fixation, knee trauma, lateral trochlea, open knee injury, osteochondral fracture, patellofemoral joint, quadriceps tendon, suprapatellar pouch, trochlear fracture

## Abstract

Open osteochondral fractures of the knee, particularly of the anterolateral trochlea, are rare and pose diagnostic and therapeutic challenges. A 42-year-old male sustained direct trauma to the left knee, right ankle, and elbow, with imaging confirming an open osteochondral fracture of the lateral trochlea and a detached fragment in the suprapatellar pouch. Surgical debridement and fixation with Herbert screws were performed. Fibrocartilage identified at the quadriceps tendon-patellar junction suggests a role in stress transmission that contributes to the fracture. Postoperative management involved early mobilization with brace protection, leading to successful healing. This case underscores the importance of timely intervention, precise fixation, and controlled rehabilitation while highlighting the need for further research into the biomechanical role of fibrocartilage in similar injuries.

## Introduction

Osteochondral fractures are commonly observed in both young and adult populations following traumatic knee injuries [1]. These fractures typically occur as a result of high-impact sports or accidents [2]. However, cases involving open osteochondral fractures are exceedingly rare in the literature. In particular, there is limited literature describing open fractures affecting the anterolateral aspect of the trochlea in adults following direct knee trauma and the appropriate management of such injury. This case report highlights a rare presentation of an open osteochondral fracture of the lateral trochlea in an adult patient, providing valuable insights into diagnosis and management strategies for such uncommon injuries.

## Case presentation

**Figure 1:**
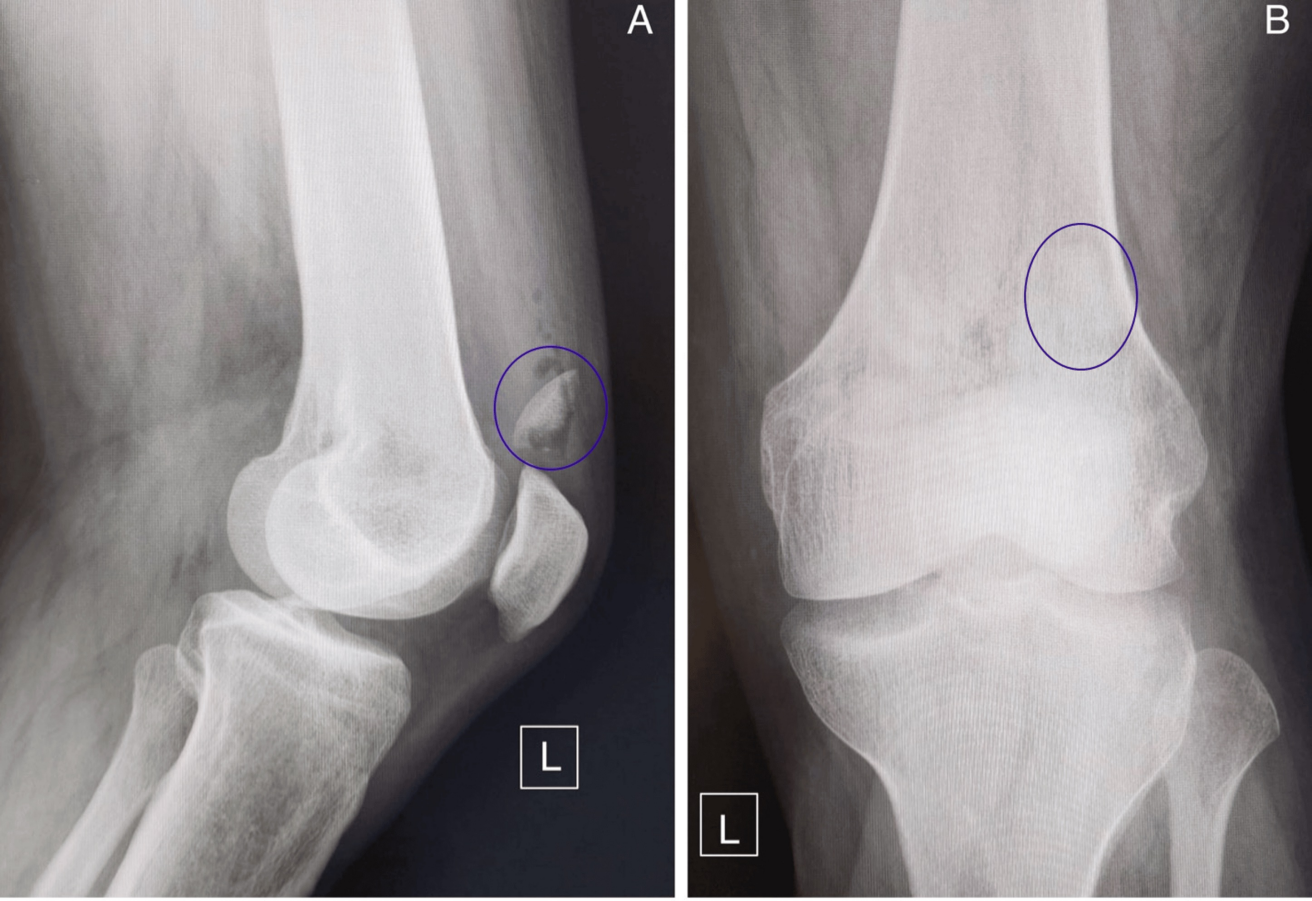
Left knee X-rays - (A) lateral and (B) anteroposterior views - obtained at initial presentation, with the osteochondral fragment highlighted by a blue circle.

**Figure 2:**
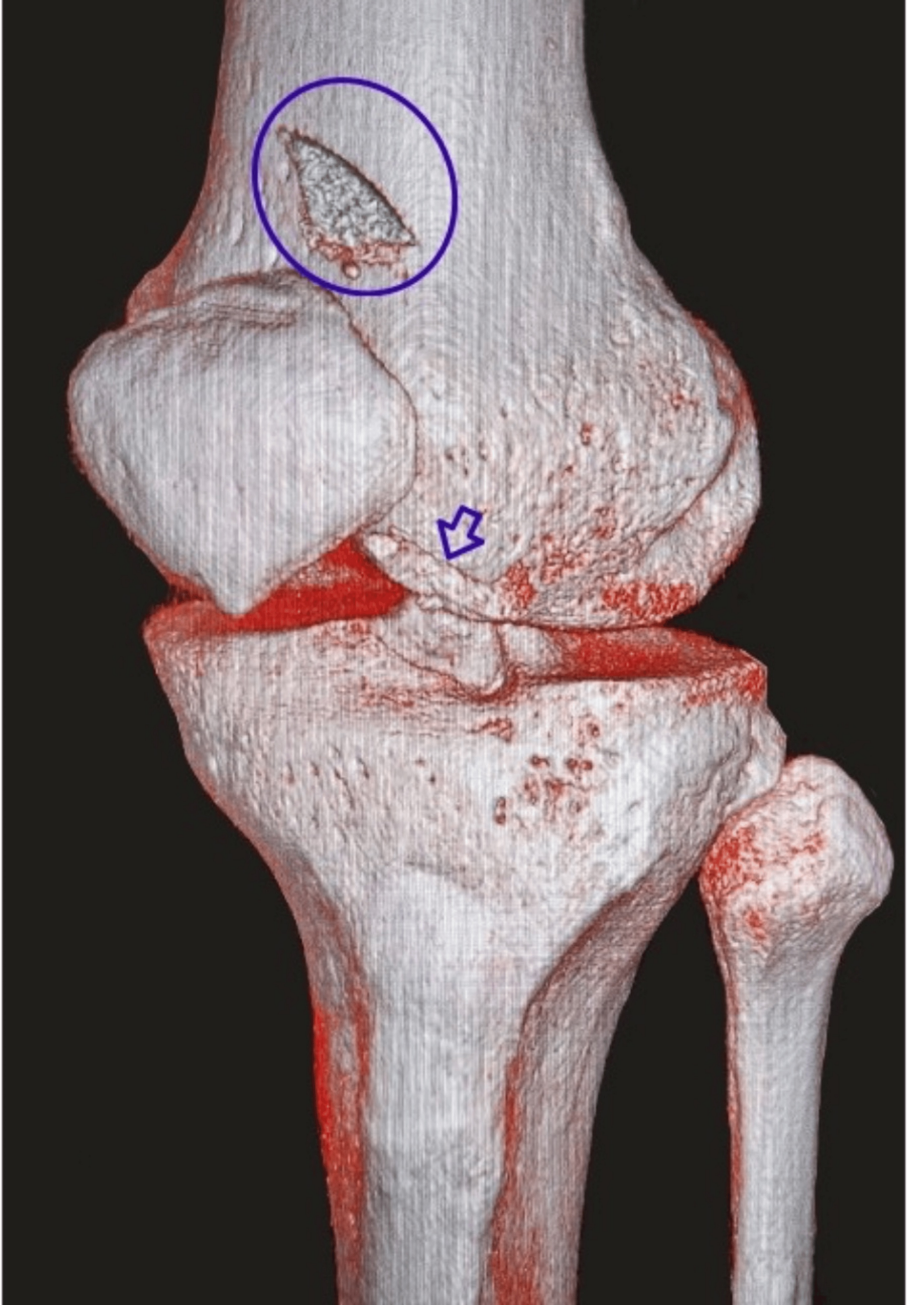
3D reconstruction from the CT scan performed at initial presentation, with the blue circle highlighting the osteochondral fragment and the blue arrow indicating the trochlear defect.

A 42-year-old male patient was brought to the emergency room (ER) by ambulance after sustaining direct trauma to the left knee, right ankle, and right shoulder. The initial evaluation by the ER physician revealed a deep wound over the anterolateral aspect of the left knee, exposing the lateral compartment of the knee joint, along with edema and severe pain at the acromioclavicular joint of the right shoulder and swelling of the right ankle. Radiographs of the ankle and shoulder demonstrated a stage II acromioclavicular disjunction, with no fractures detected in the ankle. Imaging of the left knee, including X-ray (Figure 1) and CT scan with 3D reconstruction (Figure 2), revealed an osteochondral fracture of the lateral trochlea.

**Figure 3:**
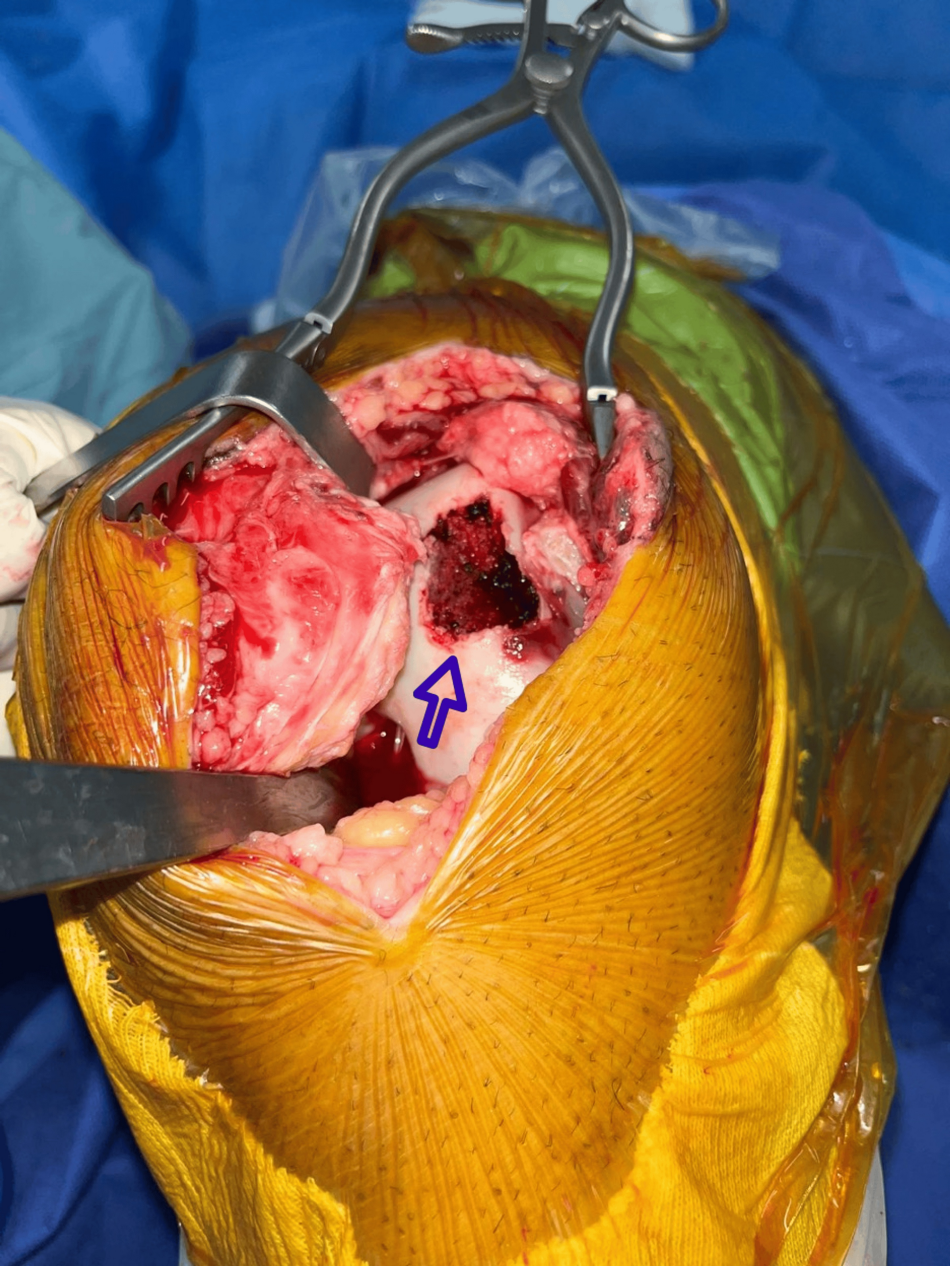
Trochlear defect (indicated by the blue arrow).

**Figure 4:**
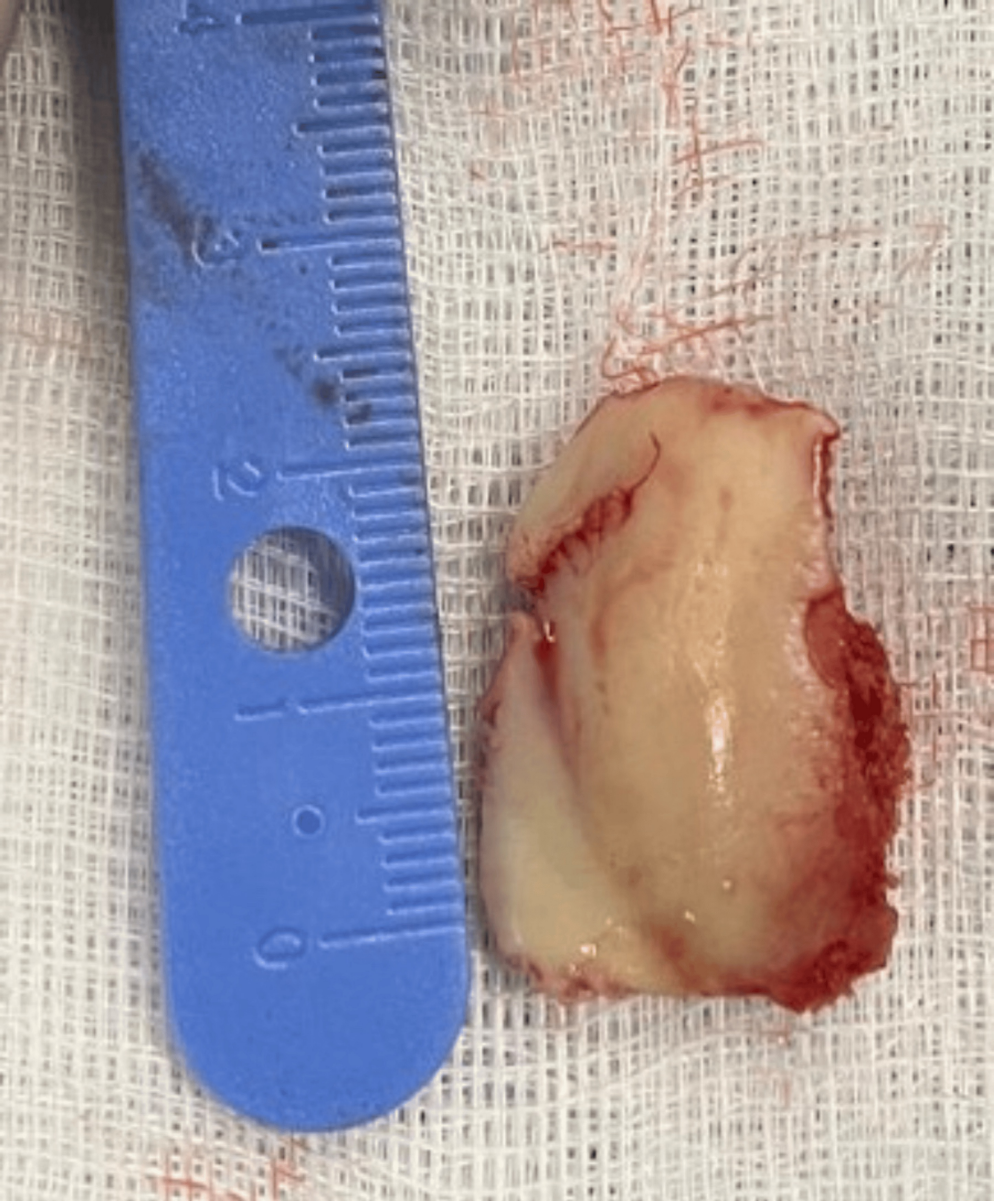
Trochlear fragment lodged in the suprapatellar pouch.

**Figure 5:**
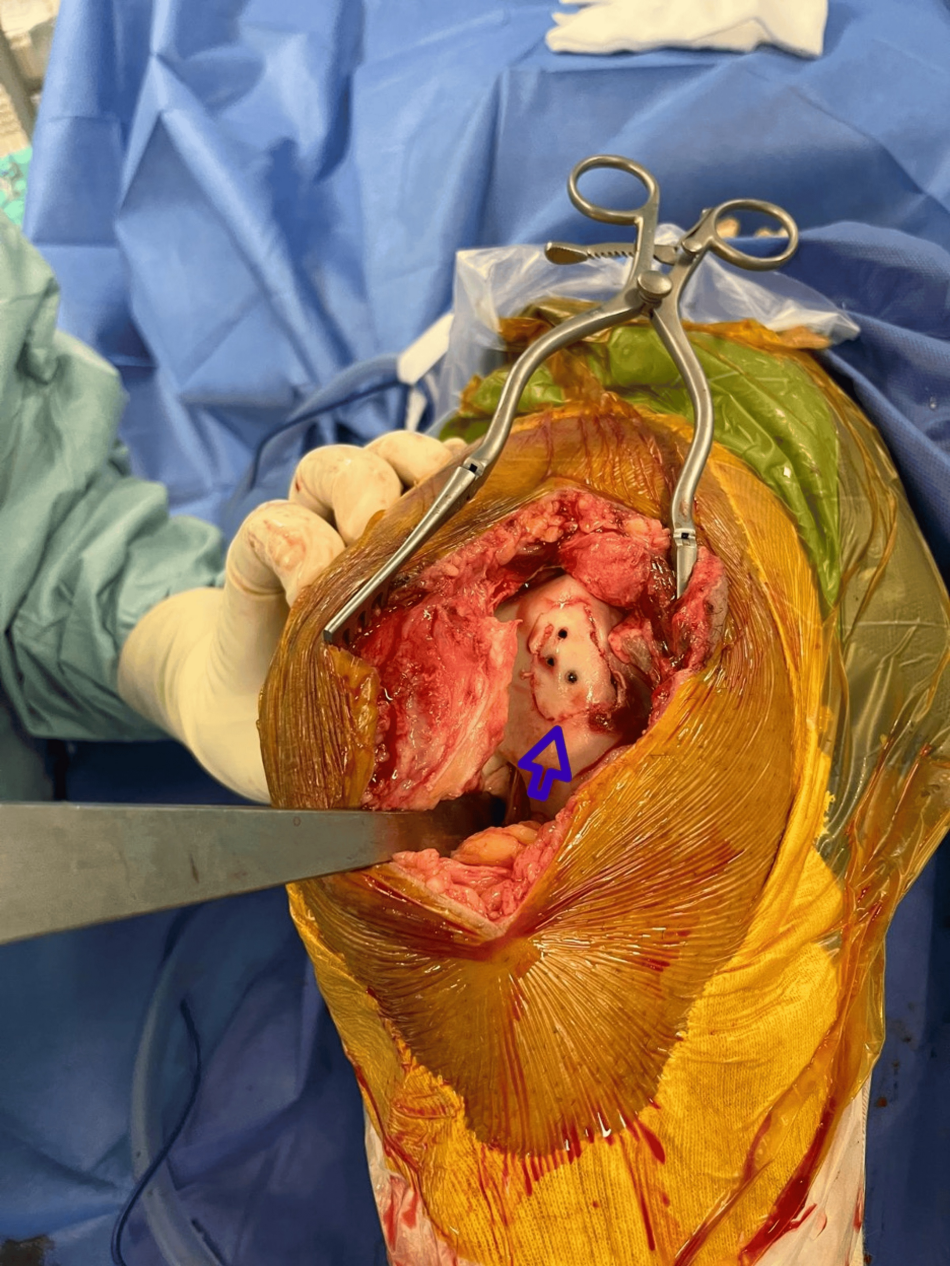
Clinical image of the trochlear fragment (indicated by the blue arrow) following fixation with three Herbert screws.

**Figure 6:**
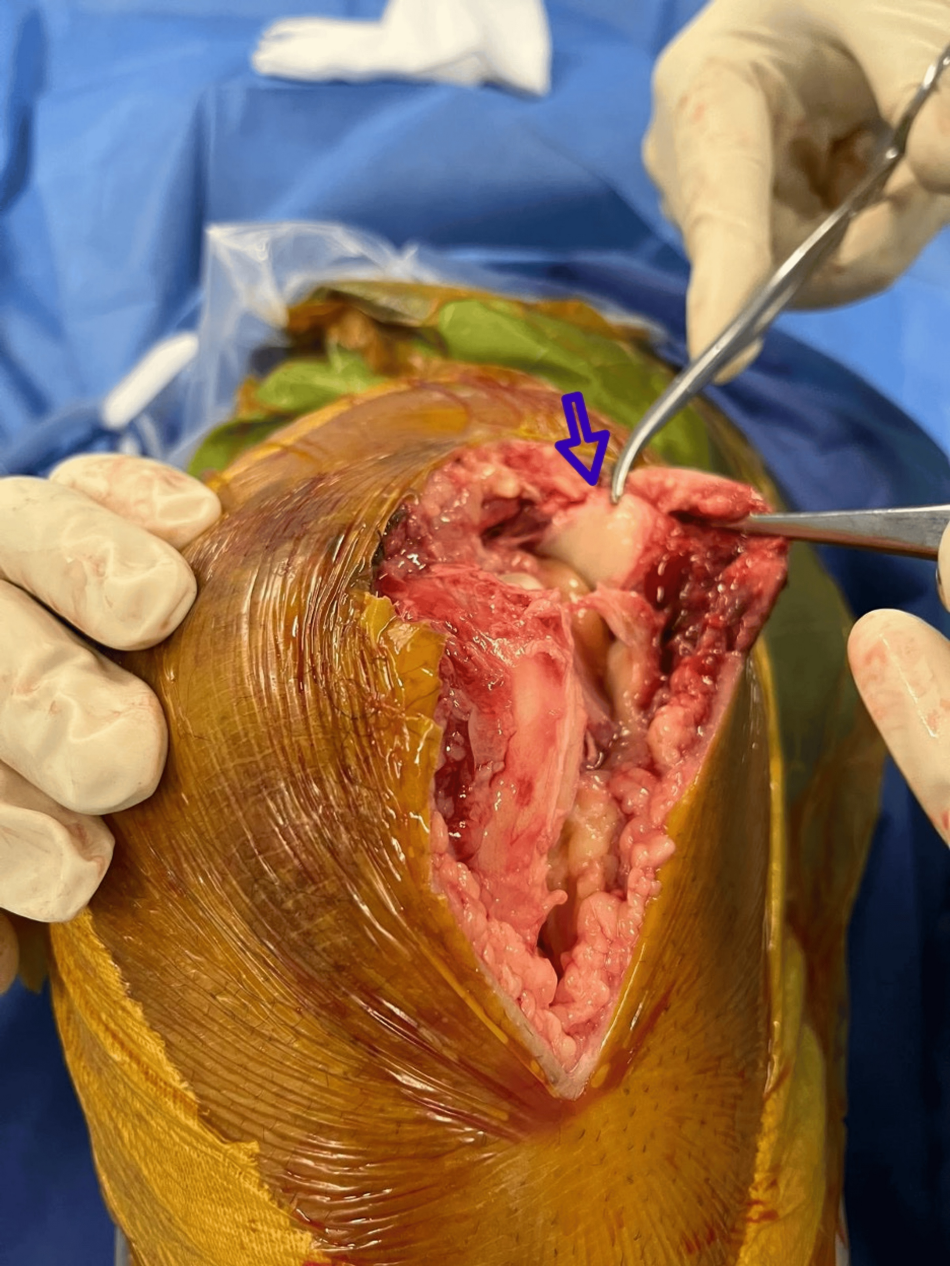
Fibrocartilage fragment (pointed out by the blue arrow) seen at the junction of the quadriceps tendon and the superior pole of the patella.

The osteochondral fragment was found to be detached and lodged in the suprapatellar pouch. The patient was administered antibiotics and tetanus prophylaxis, consent form for surgical exploration and debridement of the wound and fixation of the osteochondral fragment was signed by the patient, and he was subsequently taken to the operating room. General anesthesia was performed. Exposure of the knee joint and the trochlear defect (Figure 3) through the traumatic wound at the anterolateral aspect of the knee joint was done. Identification of the osteochondral fragment that was lodged in the suprapatellar pouch (Figure 4) and fixation using three Herbert screws (Figure 5) into the defect at the anterolateral aspect of the trochlea was done under direct visualization. It is important to note that during our surgical exploration, a fibrocartilage structure (Figure 6) was found at the junction between the quadriceps tendon and the superior pole of the patella and that the articular surface of the patella was intact.

**Figure 7:**
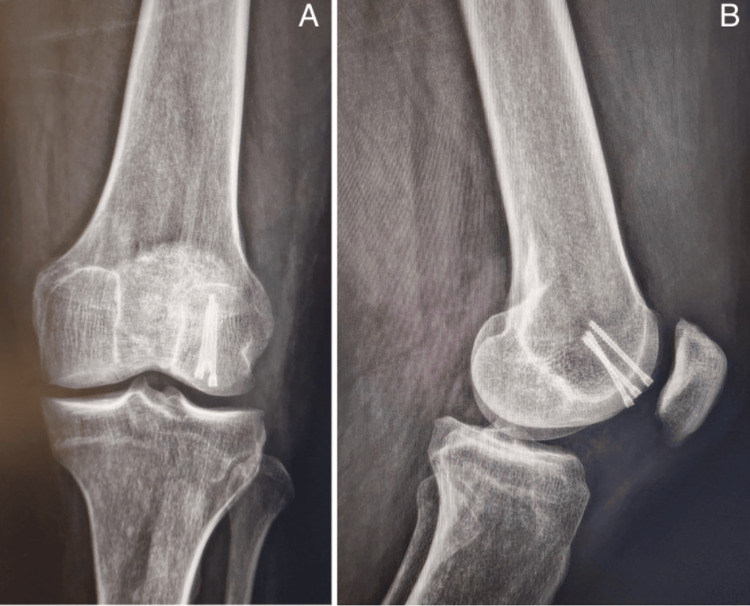
Follow-up X-ray at eight weeks post-intervention showing consolidation of the osteochondral fragment.

**Figure 8:**
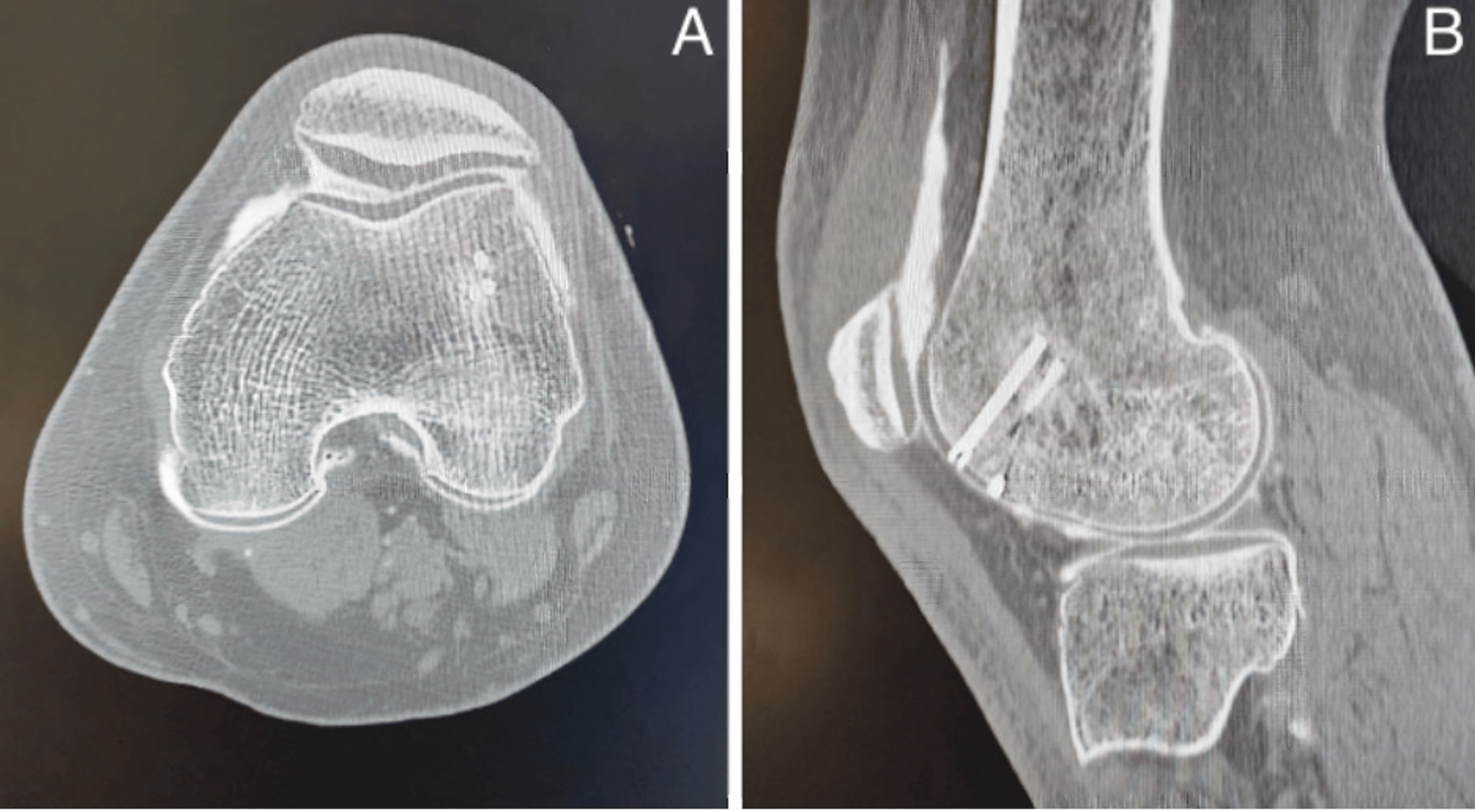
Follow-up arthro-CT scan at 1.5 years post-intervention showed consolidation of the osteochondral fragment, with no evidence of subsequent chondropathy or chondrolysis.

This finding suggests a potential involvement in the isolated trochlear fracture mechanism, as fibrocartilage at such sites is known to adapt to mechanical stress and may have played a role in force transmission leading to the lesion. Verification of satisfactory fracture reduction and hardware placement under fluoroscopy. Copious irrigation of the wound and debridement of devitalized tissue were performed, followed by layer-by-layer closure and placement of a drain within the articulation. Immobilization of the knee with a knee brace in extension was done to protect the wound repair. The patient was hospitalized for 48 hours postoperatively and discharged home thereafter. The drain was removed on day 2 (J2), and IV antibiotics were administered for 48 hours postoperatively. Full range of motion was allowed immediately in the postoperative period, and full weight-bearing was permitted with the knee kept in full extension for three weeks using a knee brace. The brace was discontinued once the patient was able to perform an active leg raise. A follow-up knee X-ray (Figure 7) at eight weeks postoperatively confirmed consolidation of the osteochondral fracture fragment. The patient resumed his professional activity as a heavy vehicle driver five months after surgery. At the 1.5-year follow-up, the patient maintained a full range of motion in the knee. However, he reported anterior knee pain during deep flexion activities, such as squatting. An arthro-CT scan (Figure 8) revealed no signs of chondrolysis or chondropathy to explain the anterior knee pain.

## Discussion

Osteochondral fractures of the trochlea, especially open fractures, present significant diagnostic and therapeutic challenges. The trochlea plays a crucial role in patellofemoral joint function, and fractures in this region can severely affect knee stability and mobility if not managed appropriately [3]. The rarity of open osteochondral fractures in this location complicates the development of standardized treatment protocols. Existing literature predominantly focuses on closed osteochondral injuries or intra-articular fractures. In a case report by Cordunianu et al. [1], trochlear osteochondral fractures resulting from sports injuries were managed arthroscopically with good outcomes. However, the open nature of the fracture in our case necessitated a direct approach through the traumatic wound, including surgical debridement and immediate fixation to prevent contamination and further cartilage damage. Furthermore, the presence of fibrocartilage at the junction of the quadriceps tendon and the superior pole of the patella is a notable finding. This fibrocartilaginous adaptation is well-documented in response to mechanical stress and compressive forces, especially at tendon-bone insertion sites known as fibrocartilaginous entheses. Histologically, such entheses consist of four distinct zones: tendon proper, uncalcified fibrocartilage, calcified fibrocartilage, and subchondral bone, forming a gradual transition that reduces stress concentration and facilitates smooth force transmission from soft tissue to bone [4]. Interestingly, the quantity and distribution of fibrocartilage in the quadriceps and patellar tendons differ. As reported by Evans et al. [5], there is more fibrocartilage at the attachment of the quadriceps tendon to the superior pole of the patella. This is attributed to the larger size of the quadriceps tendon compared to the patellar ligament, resulting in greater mechanical stress at the superior insertion. Conversely, the smaller amount of fibrocartilage at the inferior patellar pole is explained by the minimal change in the angle of force transmission at this site, reducing the need for extensive fibrocartilaginous buffering [4]. In the context of trauma, such fibrocartilaginous regions may become sites of altered force transmission, predisposing certain areas of the knee to injury. In our case, the fibrocartilage structure identified intraoperatively at the quadriceps tendon-patella junction may have contributed to the fracture mechanism by modifying the direction and distribution of forces acting on the trochlea. Under high-impact trauma, stress concentrations at this fibrocartilage interface may have redirected load vectors toward the trochlear region, potentially explaining the isolated osteochondral fracture pattern observed. This hypothesis aligns with existing studies highlighting that fibrocartilaginous adaptations can influence load-bearing mechanics and may increase susceptibility to specific types of injuries under extreme stress conditions [6]. Ju et al. [7] described an open trochlear fracture treated with Herbert screw fixation, resulting in successful fracture healing and early mobilization. This case aligns with our management strategy, emphasizing the importance of rigid internal fixation to allow early rehabilitation to preserve joint function. Our use of Herbert screws allowed for secure fixation while minimizing cartilage damage. Rehabilitation involving early weight-bearing under brace protection promotes joint mobility while safeguarding the repair. While the literature on open osteochondral fractures of the trochlea remains limited, this case contributes to the growing evidence supporting immediate surgical intervention. Future studies and case series are essential to refine treatment protocols and improve outcomes for patients with similar injuries. Early diagnosis and accurate imaging play pivotal roles in managing osteochondral fractures. Hsu et al. [8] highlight that delayed intervention can lead to poor cartilage healing, resulting in chronic pain, instability, and increased risk of osteoarthritis. In our case, prompt imaging and rapid surgical intervention facilitated favorable outcomes, as evidenced by postoperative imaging demonstrating fracture consolidation. Our experience underscores the value of a multidisciplinary approach, incorporating orthopedic surgery, radiology, and physical therapy to ensure comprehensive care. Rehabilitation involving early weight-bearing under brace protection promotes joint mobility while safeguarding the repair. While the literature on open osteochondral fractures of the trochlea remains limited, this case contributes to the growing evidence supporting immediate surgical intervention. However, as this report reflects a single case, the ability to generalize the findings to broader patient populations is inherently limited. Although the management strategy and outcomes were favorable in this instance, further studies and case series are essential to validate and refine treatment protocols and improve outcomes for patients with similar injuries. 

## Conclusions

This case highlights the rare occurrence of an open osteochondral fracture of the lateral trochlea and the importance of timely diagnosis and surgical intervention. The presence of fibrocartilage at the quadriceps tendon-patellar junction suggests a possible role in the fracture mechanism, emphasizing the need for further investigation into biomechanical factors contributing to isolated trochlear injuries. The successful outcome in this patient demonstrates the value of thorough wound debridement, accurate fragment fixation, and early mobilization in preserving joint function and promoting fracture healing. As more cases are documented, clearer guidelines and protocols can be developed to enhance the management of similar injuries, ultimately improving patient care and long-term outcomes.
